# Relationship of Serum Betatrophin with Nonalcoholic Fatty Liver in a Chinese Population

**DOI:** 10.1371/journal.pone.0170758

**Published:** 2017-01-26

**Authors:** Wen Hu, Xiaojuan Shao, Dianxuan Guo, Hairong Hao, Yong Zhang, Mingfeng Xia, Yingyun Gong, Hongwen Zhou, Yunqing Fan, Weinan Yu

**Affiliations:** 1 Department of Endocrinology, Huai'an Hospital Affiliated to Xuzhou Medical College, and Huai'an Second People's Hospital, Huai'an, Jiangsu, China; 2 Department of Geriatrics, Huai'an Hospital Affiliated to Xuzhou Medical College, and Huai'an Second People's Hospital, Huai'an, Jiangsu, China; 3 Department of Endocrinology, Zhongshan Hospital Affiliated to Fudan University, Shanghai, China; 4 Department of Endocrinology, The First Affiliated Hospital of Nanjing Medical University, Nanjing, Jiangsu, China; 5 Department of Color Doppler Ultrasound, Huai'an Hospital Affiliated to Xuzhou Medical College, and Huai'an Second People's Hospital, Huai'an, Jiangsu, China; The Chinese University of Hong Kong, HONG KONG

## Abstract

**Objective:**

This study aimed to investigate the association of serum betatrophin with the status and progression of nonalcoholic fatty liver disease (NAFLD).

**Methods:**

A total of 249 subjects who received ultrasonic examination of liver fat content (LFC) were recruited. Anthropometric and biochemical examinations were performed. Serum betatrophin was measured by ELISA.

**Results:**

Compared with control group, serum betatrophin significantly increased in NAFLD group (*P* < 0.05). There was significant difference in serum betatrophin among control, low liver fat content (LLFC), and high liver fat content (HLFC) groups (*P* < 0.01). After adjustment for gender, age, BMI, FPG and HbA1c, the betatrophin positively correlated with LFC (r = 0.185, *P* < 0.01) and TG (r = 0.195, *P* < 0.01). Stepwise multiple regression analysis indicated serum betatrophin was independently related to LFC (*P* < 0.05). Multivariate logistic regression analysis revealed subjects in the highest tertile of serum betatrophin had higher odds of having NAFLD after adjustment for traditional NAFLD risk factors (OR = 2.88, 95%CI: 1.15–7.19) (P<0.05).

**Conclusion:**

Serum betatrophin is an independent risk factor for NAFLD and potential non-invasive marker for its progression. Serum betatrophin may be helpful for the early diagnosis of NAFLD and improvement of its prognosis.

## Introduction

Nonalcoholic fatty liver disease (NAFLD) has become a worldwide public health problem affecting nearly a third of global population [1, 2]. NAFLD has a higher prevalence in China and its incidence is approximately 15% in adults of Shanghai, Guangzhou, Hong Kong and other developed areas [3]. It encompasses a spectrum ranging from non-alcoholic fatty liver (NAFL) to non-alcoholic steatohepatitis (NASH), fibrosis, cirrhosis and hepatocellular carcinoma (HCC) [4]. There is evidence showing that NAFL is a stable disease regardless of slow histologic progression [5]. However, NASH is a progressive disease and may further develop into cirrhosis or even HCC [6, 7].

At present, liver biopsy is still the gold standard for the diagnosis of NAFLD and the evaluation of its progression [8]. However, it is invasive and has some disadvantages such as high cost, insufficient sampling tissues, complications (such as bile leakage), sampling error and others [9]. Thus, it is not recommended as a technique in the population screening. In clinical practice, clinical imaging examinations such as ultrasonography (US), computerized tomography (CT) and magnetic resonance imaging (MRI) are also able to identify increased fat accumulation in the liver parenchyma. CT has higher sensitivity and specificity in the diagnosis of middle and severe hepatic steatosis, but it is not suitable for patients with mild hepatic fat deposition [10]. MRI is more sensitive than CT in the detection of hepatic steatosis, but, it is not reliable in the measurement of fat content [11]. US is widely used for the screening of NAFLD due to its easy availability, simplicity, low cost and no radiation. However, the results of US are operator-dependent and there is significant intra- and interobserver variability [12]. Moreover, it is qualitative technique and may not quantify the degree of fat accumulation. Therefore, clinical imaging examination may not be used to evaluate the liver function in NAFLD patients as well as the liver inflammation and fibrosis. Thus, it is imperative to develop new clinical non-invasive markers for the identification of individuals with high risk for NASH in NAFLD patients, the prediction of its progression and the early intervenetion.

Betatrophin is a novel active peptide that is secreted primarily from adipose tissues and the liver [13]. Yi et al found that betatrophin dramatically promoted the proliferation of pancreatic islet beta cells, increased functional beta cells mass over time and improved glucose tolerance in mice [14]. Moreover, rencent reports show that betatrophin is likely to inhibit lipoprotein lipase (LPL) activity by decomposing angiopoietin-like protein 3 (ANGPTL3) and releasing the N-terminal domain, which, in turn, inhibits LPL. LPL activity inhibition weakens the clearance of triglycerides (TG), and eventually dramatically increases serum TG in mice [15]. Therefore, betatrophin is likely to regulate glucose homeostasis and lipid metabolism in mice. The 'two-hit' hypothesis [16] has been proposed in the pathogenesis of NAFLD: in the first hit, the triglycerides accumulated in hepatocytes promote the development of hepatic steatosis; in the second hit, oxidative stress and peroxidation lead to mitochondrial dysfunction, and then hepatic stellate cell proliferate and induce hepatocyte injury, inflammation, and fibrosis. Therefore, we speculate that betatrophin plays an important role in the occurrence and development of NAFLD. However, few studies focus on the role of betatrophin in the occurrence of NAFLD and its progression.

In our study, the improved US quantitative method was employed, in which computer was combined with ultrasound images to evaluate the liver fat content (LFC) [17]. Xia et al [18] found that the new method could effectively avoid the subjective bias of the traditional ultrasound operator by measuring objective data, relatively accurately estimate LFC and detect mild hepatic steatosis with higher sensitivity. Therefore, the present study was undertaken to investigate the association between serum betatrophin and LFC detected with US quantitative method in a Chinese population, aiming to evaluate the role of betatrophin in the prediction of NAFL and its progression.

## Patients and Methods

### Patients

A total of 300 subjects who received detection of LFC by ultrasonography were recruited from the Huai’an Diabetes Prevention Program (ChiCTR-TRC-14005029) between May 2015 and July 2015 at the Health Examination Center of Huai’an Second Hospital, Affiliated Hospital of Xuzhou Medical College in Huaian (Jiangsu, China). Exclusive criteria were as follows: (1) patients had excess alcoholic drinking, (>20g/day for men and >10g/day for women) (n = 16); (2) patients were medicated with drugs known to induce steatosis; (3) patients had viral hepatitis (HBV or HCV), rare autoimmune liver disease, Wilson disease, α1-antitrypsin deficiency, liver malignant tumor, infection or biliary diseases (n = 15); (4) the ultrasonic images were unclear (n = 20). Finally, 249 subjects (120 males) were included for analysis.

According to previously reported [18], optimal cut-off value in the diagnosis NAFLD by ultrasonic detection of LFC is 9.15%, and in our study, the median LFC is 20%. In this study, subjects were divided into three groups according to LFC: (1) Control group: LFC < 9.15% (n = 84); (2) low liver fat content (LLFC): 9.15% ≤ LFC ≤ 20% (n = 82); (3) high liver fat content (HLFC): LFC > 20% (n = 83). There were no significant differences in the age and sex among these groups.

The study was conducted in accordance with the guidelines and regulations of the 1975 Declaration of Helsinki. This study was approved by the Ethics Committee of Huaian Second Hospital, XuZhou Medical University and written informed consent was signed and obtained from all the participants.

### Data collection

The demographic characteristics, medical history, medication history, and history of smoking and drinking were recorded via questionnaire in a blind manner. The height and weight were measured for the calculation of body mass index (BMI) (weight in kilograms divided by the square of the height in meters). Blood pressure was measured after resting for 10 mins. After overnight fasting (at least 10h), blood samples were collected from the antecubital vein between 8:00 and 10:00 am. Serum alanine transaminase (ALT), aspartate aminotransferase (AST), total cholesterol (TC), triglyceride (TG), low-density lipoprotein cholesterol (LDL-C), high-density lipoprotein cholesterol (HDL-C), fasting glucose (FPG), creatinine (CREA), urea nitrogen (BUN), and uric acid (UA) were measured in a certified laboratory with standardized procedures. HbA1c was measured by high performance liquid chromatography (Variant II and D-10 Systems, Bio-Rad Laboratories Inc., Hercules, CA, USA).

Serum betatrophin was measured using a commercially available human ELISA kit (Beijing Cheng Lin biological technology co, LTD, Beijing, China). Detection was carried out according to the manufacturers’ instructions [15, 19]. The optical density (OD) measured at 450 nm wave size is proportional to the concentration of betatrophin. Thus, the concentration of betatrophin was calculated on the basis of standard curve delineated according to OD. The lower and upper limits of detection were 1.5 and 60 ng/mL, respectively. The difference in intra-assay and inter-assay is less than 9% and 11%, respectively.

Hepatic ultrasonography was performed in all participants by a trained expert using an ALOKA Prosound a6 scanner (HITACHI ALOKA, Japan) with a 3-MHz probe. The parameters used were as follows: gain was 77dB and depth was 15cm, which were standardized using a tissue-mimicking phantom before measurement. The ultrasound hepatic/renal echointensity ratio (H/R) and ultrasound hepatic echo-intensity attenuation rate (HA) were obtained from ordinary ultrasound images using the NIH-image software (Image J 1.41o, National Institutes of Health, USA). LFC was measured according to previously reported [20].

### Statistical analysis

All statistical analyses were performed using SPSS version 22.0 (SPSS Inc, Chicago, IL). Each variable was tested for normal distribution using the Kolmogorov-Smirnov test. Non-normally distributed variables were analyzed with Mann-Whitney U-test for two groups and Kruskal—Wallis test for three groups. Data are presented as a percentage, median (25th and 75th percentiles) or mean±standard deviation (SD). FPG, HbA1C, TG, ALT, AST and BUN were log transformed due to their non-normal distribution for correlation. The Student’s t-test was used for comparing normally distributed variables and the Mann-Whitney U-test for comparing non-normally distributed variables. Chi-square test was used for comparisons of categorical variables. Multiple comparisons among three groups were done with Kruskal—Wallis test. Relationships of betatrophin with liver enzymes, blood glucose, lipids and renal function were evaluated by calculation of partial correlation coefficients. A stepwise multiple regression analysis was done to find the independent variables associated with LFC. The association of betatrophin with NAFLD was estimated using multivariate logistic regression analysis in three models described below. A value of *P*< 0.05 was considered statistically significant.

## Results

### Characteristics of study population

**Fig 1:**
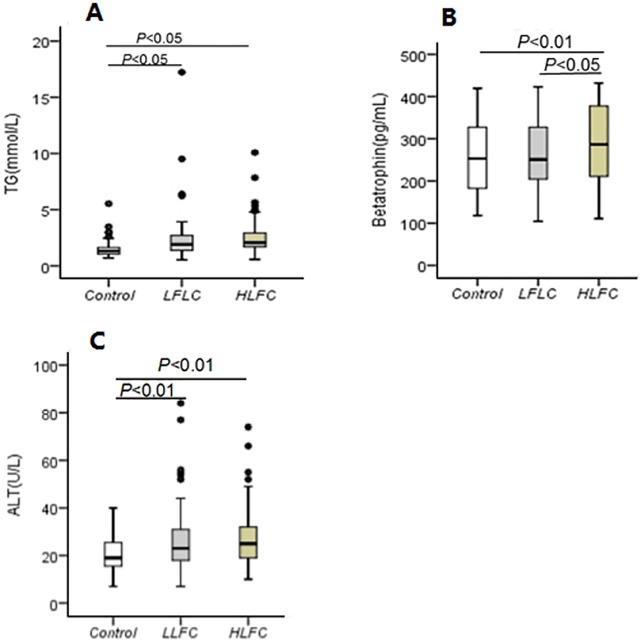
Serum betatrophin, TG and ALT in subjects according to LFC. (A) TG, triglyceride; (B) Betatrophin; (C) ALT, alanine transaminase. (A) TG and (C) ALT were significantly higher in patients with LLFC and HLFC than in controls. (B) Betatrophin was significantly higher in patients with HLFC than in patients with LLFC and in controls. An increasing trend in betatrophin was observed in patients with LLFC. Boxes represent the interquartile range between first and third quartiles, and the line (square) inside represents the median (mean).

The clinical characteristics of subjects (Control and NAFLD) at baseline are shown in Table 1. Among 249 subjects, there were 165 age- and sex- matched individuals who were diagnosed with NAFLD due to LFC higher than 9.15%. There were no significant differences in the gender and age between two groups (*P*>0.05). Compared with the control group, the DBP, serum FPG, TG, ALT, and AST in NAFLD group were increased signficantly (*P*<0.01 or *P*<0.05), while HDL-C decreased markedly (*P*<0.01). Serum betatrophin was significantly higher in NAFLD patients than in controls (*P*<0.05). Then, subjects were divided into three groups, LFC<9.15% (Control), 9.15%≤LFC≤20% (LLFC), LFC>20% (HLFC), (Fig 1). Subjects in HLFC group with more LFC had higher serum betatrophin, TG and ALT as compared to subjects in LLFC group and control group.

**Table 1:** Characteristics of subjects in this study.

Variables	Control (n = 84)	NAFLD(n = 165)	P-value
	LFC<9.15%	LFC≥9.15%	
Age (years)	60.43±0.705	59.73±0.421	0.098
Male gender, n(%)	43 (51.19)	7 (46.67)	0.500
Smoking, n(%)	14 (16.67)	32 (19.39)	0.601
SBP (mmHg)	129.44±1.60	136.52±1.31	0.133
DBP (mmHg)	80.43±0.93	83.91±0.82	0.019
BMI (kg/m^2^)	23.56±0.31	26.50±0.21	0.495
FPG (mmol/L)*	5.08 (4.76–5.5)	5.34 (4.92–5.86)	0.019
HbA1C(%)*	5.10 (4.80–5.57)	5.1 (4.80–5.50)	0.720
TC (mmol/L)	4.82±0.10	4.94±0.08	0.198
HDL-C (mmol/L)	1.58±0.04	1.34±0.02	<0.001
LDL-C (mmol/L)	2.71±0.08	2.80±0.06	0.302
TG (mmol/L)*	1.3 (1.0–1.6)	2.0 (1.6–2.8)	<0.001
ALT (U/L)*	19 (15–26)	25 (18–32)	<0.001
AST (U/L)*	21 (18–24)	22 (20–26)	0.012
CREA (μmol/L)	71.49±1.83	70.08±1.21	0.360
BUN (mmol/L)*	5.57 (4.56–6.47)	5.29 (4.66–6.15)	0.243
UA (μmol/L)	302.11±9.96	335.22±6.78	0.082
Betatrophin (pg/mL)*	252.95 (182.74–327.51)	265.64 (205.51–364.04)	0.035

All variables are expressed as n(%) for categorical data or as mean±SD or *medians (25th and 75th percentiles) for continuous data with or without a normal distribution, respectively. SBP, systolic blood pressure; DBP, diastolic blood pressure; BMI, body mass index; FPG, fasting glucose; TC, totalcholesterol; HDL-C, high-density lipoprotein cholesterol; LDL-C, low-density lipoprotein cholesterol; TG, triglyceride; ALT, alanine transaminase; AST, aspartate aminotransferase; CREA, creatinine; BUN, urea nitrogen; UA, uric acid; NAFLD, nonalcoholic fatty liver disease.

### Correlation between serum betatrophin and traditional risk factors for NAFLD

As shown in Table 2, the associations of serum betatrophin with various clinical and biochemical parameters were investigated. In partial correlations analysis, serum betatrophin was positively associated with TG (*r* = 0.168, *P* = 0.008) and LFC (*r* = 0.213, *P* = 0.001), which remained after adjustment for age, sex, BMI, FPG and HbA1c.

**Table 2:** Correlations of circulating betatrophin with clinical parameters.

Variables	Betatrophin*		Betatrophin (adjustment for age, sex, and BMI)		Betatrophin (adjustment for age, sex, BMI, FPG, HbA1c)	
	r	P-value	r	P-value	r	P-value
Age	-0.035	0.578				
BMI	0.076	0.231				
SBP	0.111	0.081	0.079	0.213	0.085	0.184
DBP	0.086	0.175	0.089	0.165	0.093	0.145
FPG*	0.066	0.297	0.098	0.126		
HbA1c*	0.069	0.280	0.099	0.120		
TC	0.046	0.473	0.082	0.202	0.080	0.213
HDL-c	-0.085	0.184	-0.013	0.840	-0.009	0.888
LDL-c	-0.041	0.524	-0.027	0.676	-0.024	0.707
TG*	0.168	0.008	0.207	0.001	0.195	0.002
ALT*	0.032	0.614	-0.017	0.786	-0.026	0.685
AST*	0.055	0.385	0.002	0.975	-0.004	0.945
CREA	0.046	0.473	-0.016	0.805	-0.005	0.943
BUN*	0.004	0.954	0.023	0.716	0.016	0.802
UA	0.042	0.510	0.014	0.830	0.023	0.723
LFC	0.213	0.001	0.191	0.003	0.185	0.004

* Log-transformed variables. BMI, body mass index; SBP, systolic blood pressure; DBP, diastolic blood pressure; FPG, fasting glucose; TC, totalcholesterol; HDL-C, high-density lipoprotein cholesterol; LDL-C, low-density lipoprotein cholesterol; TG, triglyceride; ALT, alanine transaminase; AST, aspartate aminotransferase; CREA, creatinine; BUN, urea nitrogen; UA, uric acid; LFC, liver fat content.

### Association of serum betatrophin with LFC

As shown in Table 3, stepwise multiple regression analyses showed that TG (*β* = 0.152, *P* = 0.082), BMI (*β* = 0.158, *P* = 0.013), CREA (*β* = -0.170, *P* = 0.008), HDL-C (*β* = -0.140, *P* = 0.043) and serum betatrophin (*β* = 0.152, *P* = 0.009) were independently related to LFC.

**Table 3:** Association of clinical and biochemical parameters with LFC.

LFC			
	β	F	P-value
Age	—	2.360	—
BMI	0.158	7.514	0.013
SBP	—	6.846	—
ALT	—	5.123	—
CREA	-0.170	4.987	0.008
FPG	—	4.294	—
TG	0.152	4.815	0.082
HDL-C	-0.140	4.662	0.043
Betatrophin	0.152	4.898	0.009
R^2^	0.118		0.043

*F* value was set at 4.0 at each step. BMI, body mass index; SBP, systolic blood pressure; FPG, fasting glucose; HDL-C, high-density lipoprotein cholesterol; TG, triglyceride; ALT, alanine transaminase; CREA, creatinine; LFC, liver fat content.

### Prediction of NAFLD with serum betatrophin

As shown in Table 4, after adjustment for age and gender (model 1), subjects in the highest tertile of serum betatrophin were more likely to have NAFLD (OR = 2.31, 95% CI = 1.14–4.64) (*P* = 0.019). After adjustment for BMI, FPG, TC, TG, LDL-C, and HDL-C, the correlation remained. Compared to subjects in the lowest tertile of serum betatrophin, those in the highest tertile had higher odds of having NAFLD (OR = 2.74, 95% CI = 1.13–6.62) (*P* = 0.025). After adjustment for ALT, AST, CREA, UA, BUN, SBP and DBP, subjects in highest tertile of serum betatrophin still had higher odds of having NAFLD (OR = 2.88, 95% CI = 1.15–7.19) (*P* = 0.024).

**Table 4:** Correlation of NAFLD with betatrophin.

Betatrophin* (pg/mL)	T1,n = 82 NAFLD,n = 48	T2,n = 85 NAFLD,n = 55		T3,n = 82 NAFLD,n = 62	
	170.923 (104.27–215.89)	264.893 (216.15–319.93)		370.243 (321.52–431.86)	
Models					
NAFLD	Reference	OR (95% CI)	P-value	OR (95% CI)	P-value
Model 1	1	1.34 (0.70–2.54)	0.374	2.31 (1.14–4.64)	0.019
Model 2	1	1.23 (0.54–2.79)	0.616	2.74 (1.13–6.62)	0.025
Model 3	1	1.56 (0.66–3.69)	0.315	2.88 (1.15–7.19)	0.024

Model 1: adjustment for age and gender; Model 2 adjustment for BMI, FPG, TC, TG, LDL-C, HDL-C + Model 1; Model 3 adjustment for ALT, AST, CREA, UA, BUN, SBP, DBP + Model 2. Tertiles of betatrophin are expressed as T1 (< 216.00), T2 (216.00–320.00), and T3 (> 320.00).

* Betatrophin is presented as Median (range).

## Conclusions

The present study showed that serum betatrophin was independently positively associated with TG after adjustment for a wide range of risk factors for NAFLD in a Chinese population. Serum betatrophin was an independent risk factors for LFC. Subjects with HLFC had higher serum betatrophin and TG as compared to those with LLFC and controls. Multiple logistic regression showed that the odds for NAFLD were 2.88 fold higher in third levels of serum betatrophin as compared to those in first level after adjustment for traditional risk factors of NAFLD.

Our study differed from previous investigations. Firstly, LFC was measured with the ultrasonic quantitative method. Secondly, to our knowledge, this was the first population-based study to explore the association between serum betatrophin and LFC. Thirdly, risk factors for NAFLD including glucose, lipids, BP and betatrophin are improved.

The diagnosis of NAFLD and the evaluation of its progression based on non-invasive markers are still a challenge in clinical practice. Liver biopsy as gold standard for the diagnosis of NAFLD and the evaluation of its progression is unsuitable for population based screening. Furthermore, common imaging examinations cannot determine liver function of patients with NAFLD and fail to identify the inflammation and fibrosis of fatty liver. In addition, the application of biological markers in clinical practice is still immature. Therefore, establishing a new non-invasive diagnostic method to timely identify NAFLD and evaluate its progression is imperative for its treatments and prognostic prediction.

Betatrophin/ANGPTL8, is a new adipokine secreted from hepatic or adipose tissues. It has been found to significantly stimulate pancreatic β-cell growth, increase β-cell mass and improve glucose tolerance in mouse models [14]. Betatrophin is able to inhibit LPL activity by promoting ANGPTL3 cleavage, releasing the N-terminal domain, which, in turn, inhibits LPL, dramatically increases serum TG levels in mouse models [15]. The present study showed that serum betatrophin was positively associated with TG even after adjustment for a lot of risk factors for NAFLD, which indicates that serum betatrophin is associated with the pathogenesis of NAFLD in human.

In our study, results showed LFC positively correlated with serum betatrophin even after adjustment for many risk factors. With the accumulation of LFC, patients are more likely to have NASH, which has been shown by Chalasani et al [21]. As shown in Fig 1, ALT levels were higher in HLFC patients than in LLFC subjects and in controls. The increase in ALT may prompt the progression from NAFLD to NASH to a certain extent [22]. Therefore, plasma betatrophin may indirectly reflect the progression of NAFLD, namely NASH incidence, which was consistent with the findings from the study of Arias-Loste et al [23]. In that study, a total of 55 subjects were recruited including liver cirrhosis according to histological criteria and healthy individuals, and results showed that plasma betatrophin increased in patients with cirrhosis and this increase was related to the severity of cirrhosis.

Stepwise multiple regression analysis showed that serum betatrphin was an independent factor for LFC, but the potential mechanisms remain elusive. Previous studies have shown betatrophin is able to inhibit the LPL activity by increasing TG in mice [15]. In our study, elevated serum betatrophin was also accompanied by the rise of TG in human. Therefore, we speculate that the rise of betatrophin inhibits of LPL activity in humans and decreases TG decomposition from chylomicron (CM) and very low density lipoprotein (VLDL) [24], which aggrandizes TG of CM and VLDL into hepatocytes, resulting in TG accumulation in these cell. Finally, liver steatosis occurs. Of course, more studies are required to confirm our findings. Additionally, Yong et al [25] showed that hyperlipidemia or lipotoxicity could activate endoplasmic reticulum (ER) stress and the expression of betatrophin was highly induced by ER stress in hepatocytes, however, this not confirmed in human. However, findings from a study [26] conducted in 69 patients with NAFLD and 69 healthy subjects were inconsistent with our results. This study indicated that β-trophin was lower in individuals with biopsy-proven NAFLD than in healthy controls. With the deterioration of fibrosis, serum betatrophin further reduced. The conflicting findings may be ascribed to that NAFLD subjects recruited had progressed to fibrosis and the disease was more severe than that in our study. To our knowledge, betatrophin mRNA is almost uniquely expressed in the liver [27, 28] of humans. In patients with liver fibrosis and hepatocyte necrosis, hepatocytes release a large number of inflammatory cytokines [29], which may influence the betatrophin expression. Therefore, more population based studies are required to explore the relationship between serum betatrophin and liver fibrosis.

Previous studies have shown that serum betatrophin is significantly related to plasma glucose [13, 14]. However, in our study, there were no significant correlations of betatrophin with FPG and HDL-c (P > 0.05). This may be explained as that the glucose metabolism of subjects recruited in our study was normal at baseline, therefore, serum betatrophin was not affected.

Our study also had some limitations. Firstly, this was a cross-sectional study than could not reflect causative relationship of betatrophin with NAFLD and its progression. Secondly, identification of NAFLD and the propensity to NASH are dependent on the ultrasonic method instead of liver biopsy which is the gold standard for the diagnosis and staging of NAFLD. Thirdly, subjects were limited to the same ethnicity, and studies on large populations from heterogeneous ethnicities will provide more information on the role of serum betatrophin in the pathogenesis of NAFLD and its progression.

In conclusion, the present study shows that serum betatrophin increases in NAFLD patients and is independently associated with LFC. With the rise of serum betatrophin, LFC accumulates gradually. The accumulation of fat may indirectly reflect the progression into NASH. Thus, serum betatrophin may be a potential non-invasive marker for identification of NAFLD and NASH. Further investigation is needed to investigate the role of betatrophin in the pathogenesis NAFLD and its progression.
